# Guanidinoacetic Acid Regulates Myogenic Differentiation and Muscle Growth Through miR-133a-3p and miR-1a-3p Co-mediated Akt/mTOR/S6K Signaling Pathway

**DOI:** 10.3390/ijms19092837

**Published:** 2018-09-19

**Authors:** Yujie Wang, Jideng Ma, Wanling Qiu, Jinwei Zhang, Siyuan Feng, Xiankun Zhou, Xun Wang, Long Jin, Keren Long, Lingyan Liu, Weihang Xiao, Qianzi Tang, Li Zhu, Yanzhi Jiang, Xuewei Li, Mingzhou Li

**Affiliations:** Farm Animal Genetic Resource Exploration and Innovation Key Laboratory of Sichuan Province, Chengdu 611130, China; wangyujie715@163.com (Y.W.); jideng_ma@sina.com (J.M.); qiuwanling2016@163.com (W.Q.); Jinweizhang50@163.com (J.Z.); siyuanfeng_bioinfo@163.com (S.F.); scauzxk99@163.com (X.Z.); xun_wang007@163.com (X.W.); longjin8806@163.com (L.J.); longkeren@163.com (K.L.); liulingyan_602@163.com (L.L.); weihang.xiao@sicau.edu.cn (W.X.); wupie@163.com (Q.T.); zhuli7508@163.com (L.Z.); jiangyz04@163.com (Y.J.)

**Keywords:** guanidinoacetic acid, skeletal muscle, differentiation, muscle growth, C2C12, microRNA

## Abstract

Guanidinoacetic acid (GAA), an amino acid derivative that is endogenous to animal tissues including muscle and nerve, has been reported to enhance muscular performance. MicroRNA (miRNA) is a post-transcriptional regulator that plays a key role in nutrient-mediated myogenesis. However, the effects of GAA on myogenic differentiation and skeletal muscle growth, and the potential regulatory mechanisms of miRNA in these processes have not been elucidated. In this study, we investigated the effects of GAA on proliferation, differentiation, and growth in C2C12 cells and mice. The results showed that GAA markedly inhibited the proliferation of myoblasts, along with the down-regulation of cyclin D1 (*CCND1*) and cyclin dependent kinase 4 (*CDK4*) mRNA expression, and the upregulation of cyclin dependent kinase inhibitor 1A (*P21*) mRNA expression. We also demonstrated that GAA treatment stimulated myogenic differentiation 1 (*MyoD*) and myogenin (*MyoG*) mRNA expression, resulting in an increase in the myotube fusion rate. Meanwhile, GAA supplementation promoted myotube growth through increase in total myosin heavy chain (MyHC) protein level, myotubes thickness and gastrocnemius muscle cross-sectional area. Furthermore, small RNA sequencing revealed that a total of eight miRNAs, including miR-133a-3p and miR-1a-3p cluster, showed differential expression after GAA supplementation. To further study the function of miR-133a-3p and miR-1a-3p in GAA-induced skeletal muscle growth, we transfected miR-133a-3p and miR-1a-3p mimics into myotube, which also induced muscle growth. Through bioinformatics and a dual-luciferase reporter system, the target genes of miR-133a-3p and miR-1a-3p were determined. These two miRNAs were shown to modulate the Akt/mTOR/S6K signaling pathway by restraining target gene expression. Taken together, these findings suggest that GAA supplementation can promote myoblast differentiation and skeletal muscle growth through miR-133a-3p- and miR-1a-3p-induced activation of the AKT/mTOR/S6K signaling pathway.

## 1. Introduction

Skeletal muscle, which accounts for approximately 40% of body weight in mammals, plays an important role in maintaining body balance and coordinating locomotion. Generally, skeletal muscle is composed of myofibers which constitute 50–75% of the total proteins in the body and, under physiological conditions, exhibits a state of relative homeostasis of protein synthesis and degradation. However, many external factors can disrupt this balance, including nutrition, exercise, and the addition of exogenous nutrients [1,2,3], leading to an increase in the synthesis of skeletal muscle protein. For instance, alpha-ketoglutarate can induce the synthesis of skeletal muscle protein through the Akt/mTOR signaling pathway, while betaine enhances skeletal muscle myoblast differentiation by regulating myogenic transcription factors and activating Insulin-Like growth factor 1 (IGF-1) signaling. Guanidinoacetic acid (GAA), a natural amino acid derivative, has been used as a human dietary supplement to enhance muscular performance of healthy volunteers [4] and has been suggested to have potential therapeutic use to prevent muscle mass loss [5]. Moreover, replenishment of GAA was reported to improve quality of life in patients with chronic fatigue syndrome [6] and enhance muscle production in livestock [7,8]. The above effects are probably due to GAA stimulating cellular bioenergetics by enhancing the biosynthesis of creatine [9], which is an essential compound in the energy metabolism of muscle and nerve tissue. However, the effect of GAA on skeletal muscle growth and myogenic differentiation remains unclear.

MicroRNAs (miRNAs), a large class of evolutionarily conserved non-coding RNAs in eukaryotes, regulate target gene expression by mediating mRNA degradation or inhibiting translation at the post-transcriptional level [10,11]. They have been suggested to be involved in a variety of cellular processes, including proliferation, differentiation, and apoptosis [12,13,14]. Recent studies have demonstrated that a subset of muscle-specific or muscle-enriched miRNAs termed myomiRs play vital roles in myogenesis, which has extended our understanding of the molecular regulatory networks in skeletal muscle [15]. For example, miR-1 inhibits the expression of histone deacetylase 4 (HDAC4) to facilitate myoblast differentiation. miR-133 promotes myoblast proliferation by repressing the expression of serum response factor (SRF) [16,17]. In addition, some myomiRs enhance myoblast differentiation by targeting myogenesis-associated transcription factors Pax7 and Pax3 [18,19,20], and some myomiR affect muscle growth and atrophy [21,22]. Notably, the expression of myomiRs is strictly regulated by MyoD, MyoG [20,23,24,25] to precisely regulate the plasticity of skeletal muscle. Meanwhile, miRNAs are also known to participate in nutrient-regulated myogenesis. For instance, genistein, which is abundant in soybean, enhances myogenic differentiation by regulating miR-222 expression [26], methionine induces myogenic differentiation in trout using uses miR-210 [27], and dietary energy levels affect muscle fiber type transformation by changing miRNA expression [28].

In previous studies, GAA was shown to enhance muscular athletic performance (muscle strength, anaerobic performance and aerobic performance) in humans [29] and improved growth, lean meat percentage and breast meat in domestic animals, such as chicken [7,8] and pigs [30]. However, the roles of GAA in the regulation of myogenic differentiation and growth are unclear, and the underlying mechanisms by which GAA-associated miRNAs regulate myogenesis have not been studied. Hence, we investigated the effects of GAA on skeletal muscle proliferation, differentiation, and growth, and explored the roles of miRNA in these processes.

## 2. Results

### 2.1. GAA Affects Myogenic Differentiation of Myoblast in Dose-Dependent Manner

**Figure 1:**
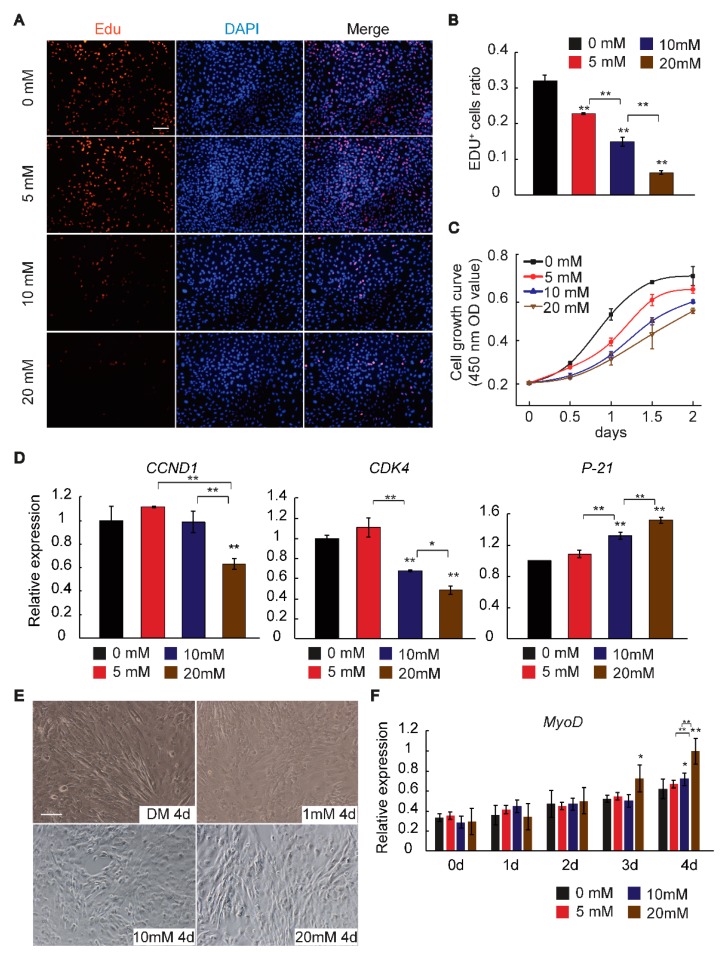
Effect of Guanidinoacetic Acid (GAA) on proliferating myoblasts. Myoblasts were cultivated in growth medium (GM) or differentiation medium (DM) in the presence of 0, 5, 10, or 20 mM GAA. (**A**,**B**) EdU analysis showed that GAA significantly reduced the cell proliferation ratio; (**C**) CCK-8 tests indicated that cell growth was suppressed by GAA in a dose-dependent manner; (**D**) relative expression of marker genes of proliferation, including Cyclin D1 (*CCND1*), Cyclin Dependent Kinase 4 (*CDK4*) and Cyclin Dependent Kinase Inhibitor 1A (*P21*) after treatment with the concentration gradient of GAA; (**E**) images were obtained by brightfield microscopy on the fourth day of myoblast differentiation after stimulation with the concentration gradient of GAA; (**F**) the relative expression of myogenic differentiation 1 (*MyoD*). The results are expressed as mean ± S.D. of three independent experiments. * *p* < 0.05; ** *p* < 0.01 compared with the negative control (on the bars) or between the indicated groups (Tukey’s post-hoc test).

In this study, we used the C2C12 line, which is a well-established cell model, for the study of muscle differentiation in vitro to explore the effect of GAA on myogenesis. C2C12 cells were cultivated in growth medium (GM) or differentiation medium (DM) containing a concentration gradient of GAA. Firstly, we tested whether C2C12 cell could utilize GAA. The creatine content was detected and it was revealed that GAA supplement increased the cellular concentration of creatine (Figure S1A). Next, cell counting kit-8 (CCK-8) and the Edu+ cell radio, as well as the expression of proliferation-related genes, including Cyclin D1 (*CCND1*) [31], Cyclin Dependent Kinase 4 (*CDK4*) [32] and Cyclin Dependent Kinase Inhibitor 1A (*P21*) [33], suggested that GAA inhibited C2C12 cell proliferation in a dose-dependent manner (Figure 1A–D). The authors also detected the effects of GAA on the differentiation phase of the first four days. As as shown in Figure 1E,F, GAA increased myotube length and enhanced Myogenic Differentiation 1 (*MyoD*) expression in a dose-dependent manner. These findings imply that GAA can reduce the myoblast proliferation ratio and enhance the early differentiation of myoblasts in a dose-dependent manner.

**Figure 2:**
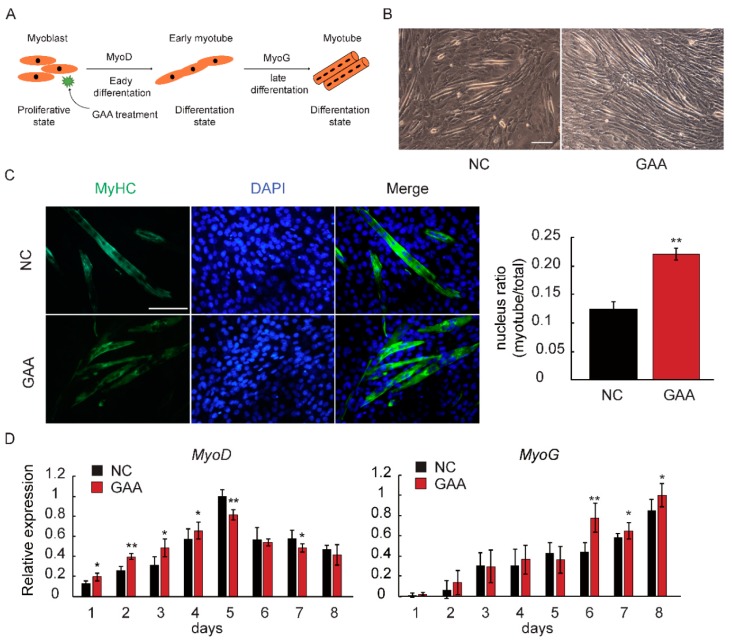
Effect of GAA on myoblasts during differentiation. (**A**) An overview of the experimental procedures; (**B**,**C**) myoblasts were induced to differentiate in the presence or absence of 20 mM GAA for 6 days. Images were obtained by brightfield microscopy on the 6th day of myoblast differentiation after 20 mM GAA treatment. Meanwhile, cells were stained with anti-MyHC antibody and the fusion index was calculated (Student’s *t*-test); negative control (NC) means a cell undergoes normal differentiation process; (**D**) the relative expression of marker genes of skeletal muscle differentiation (early phase: *MyoD*, later phase: *MyoG*) was examined by quantitative real-time PCR (Student’s *t*-test). The results are expressed as the mean ± S.D. of three independent experiments. * *p* < 0.05; ** *p* < 0.01 compared with the negative control on the bars).

MyoD is involved at an early stage of myogenesis to determine myogenic fate, while Myogenin (MyoG) acts at a later phase to control myoblast fusion [34,35]. We used differentiating myoblasts treated with 20 mM GAA for 8 days to further study the effect of GAA on myoblasts at different phases of differentiation (Figure 2A). Brightfield microscopy results revealed that GAA treatment clearly induced an elongated myoblast morphology (Figure 2B). Subsequently, immunofluorescence data revealed that the myosin heavy chain-positive (MyHC) myotubes and nuclear ratio were significantly increased upon exposure to GAA (Figure 2C). Meanwhile, the qRT-PCR results indicated that the expression of *MyoD* and *MyoG* was enhanced by GAA treatment (Figure 2D).

The above results suggest that GAA treatment adversely affected the myoblast proliferation phase. In contrast, the GAA-mediated morphological changes, such as improved fusion rate, as well as increased *MyoD* and *MyoG* levels, were consistent with the effect of the nutrient in promoting myoblast differentiation.

### 2.2. GAA Modulates Myogenesis through MicroRNA Regulatory Network

**Figure 3:**
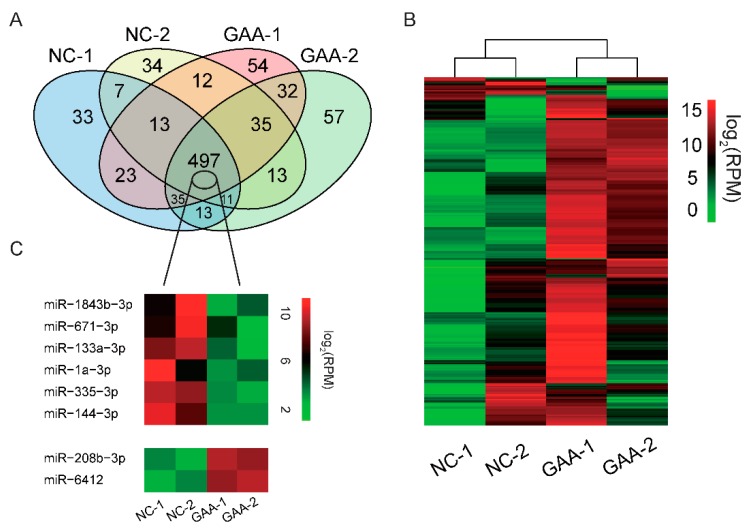

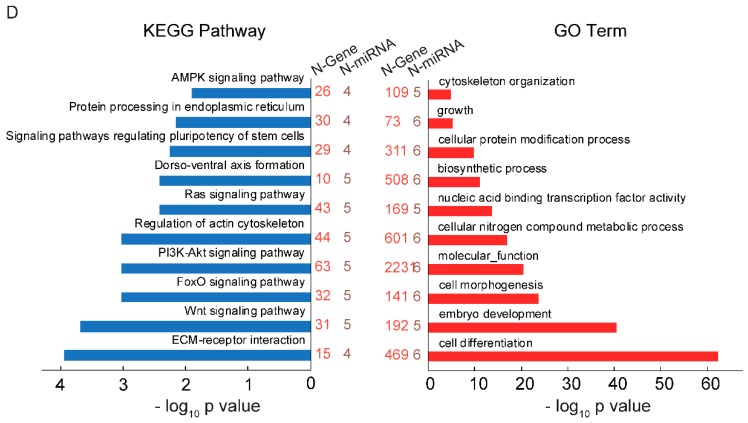
GAA modulates miRNA expression profile. Myoblasts were placed in DM with or without 20 mM GAA; 6 days later, total RNA was collected and small RNA-seq was carried out. (**A**) Venn diagram showing the numbers of overlapping and unique miRNAs detected in negative control and GAA-stimulated groups; (**B**) hierarchical clustering analysis for myotubes treated with GAA; NC-1 and NC-2 are two duplicates of the negative control; GAA-1 and GAA-2 are two duplicates of the GAA control; (**C**) the DE miRNAs that overlap between the negative control and GAA control; (**D**) GO (Gene Ontology) categories and KEGG (Kyoto Encyclopedia of Genes and Genomes) pathways enriched for target genes of DE miRNAs in myotubes (N-Gene: the number of genes enriched into indicated item, N-miRNA: the number of miRNA enriched into indicated item).

Accumulating research has indicated that miRNAs play a central role in muscle development and disease [36]. It has been reported that exogenous nutrients (resveratrol and xylobiose) can modulate physiological functions by altering the miRNA expression profile [37,38]. Prompted by these findings, we explored whether miRNAs play an important role in GAA-induced myogenic differentiation. We collected myotube RNA on the 6th day after treatment with 20 mM GAA and carried out small RNA-seq. The results revealed that 14.76 M, 14.70 M, 14.12 M, and 11.42 M raw reads were generated from negative control-1, negative control-2, GAA treatment-1 and GAA treatment-2 (NC-1, NC-2, GAA-1, and GAA-2), respectively. After the raw reads had been subjected to a series of additional filters, 8.80 M, 9.68 M, 9.86 M, and 7.28 M were defined as clean reads. Subsequently, the numbers of overlapping and unique miRNAs in NC and GAA-treated myotube cells were determined, as showed in Figure 3A. Overlapping miRNAs accounted for 75.4% of the total, but the proportion of specific expression of miRNAs was low (Table S4). This indicates that specifically expressed miRNAs have a small effect on GAA-induced myogenesis. To determine whether the expression profiles of miRNAs are affected by GAA, we performed hierarchical clustering analysis of four samples. As showed in Figure 3B, the clustering accurately separated the samples into two main branches (one branch consisted of NC-1 and NC-2, and the other consisted of GAA-1 and GAA-2). This showed the repeatability of our experiment. Meanwhile, through Differential expression (DE) analysis, we identified eight co-expressed DE miRNAs (Figure 3C and Table S5). Notably, among the DE miRNAs, the expression of miR-133a-3p and the miR1a-3p cluster, which play a role in myogenesis, was downregulated. In addition, we selected 10 miRNAs, which contained DE miRNA, for validation of their expression. The results indicate a strong positive correlation between the small RNA-seq and the qRT-PCR results (Figure S2) (Spearman’s *r* = 0.608, *p* < 0.01), which emphasized the reliability of our sequencing data. The above results matched expectations, with GAA being shown to affect the miRNA expression profile of myotubes.

To further understand the potential functions of the DE miRNAs, we carried out target prediction and functional enrichment analysis. As expected, the target genes of the downregulated miRNAs were associated with processes of myogenesis, such as cell differentiation (469 genes, *p* = 3.64 × 10^−63^), Wnt signaling pathway (31 genes, *p* = 1.15 × 10^−4^), PI3K–Akt signaling pathway (63 genes, *p* = 9.45 × 10^−4^), and AMPK signaling pathway (26 genes, *p* = 0.012) (Figure 3D). These results suggest that exposure of cells to DM containing GAA promotes their ability to differentiate into myotubes.

### 2.3. MiR-133a-3p and MiR-1a-3p Expression is Decreased during Muscle Growth

**Figure 4:**
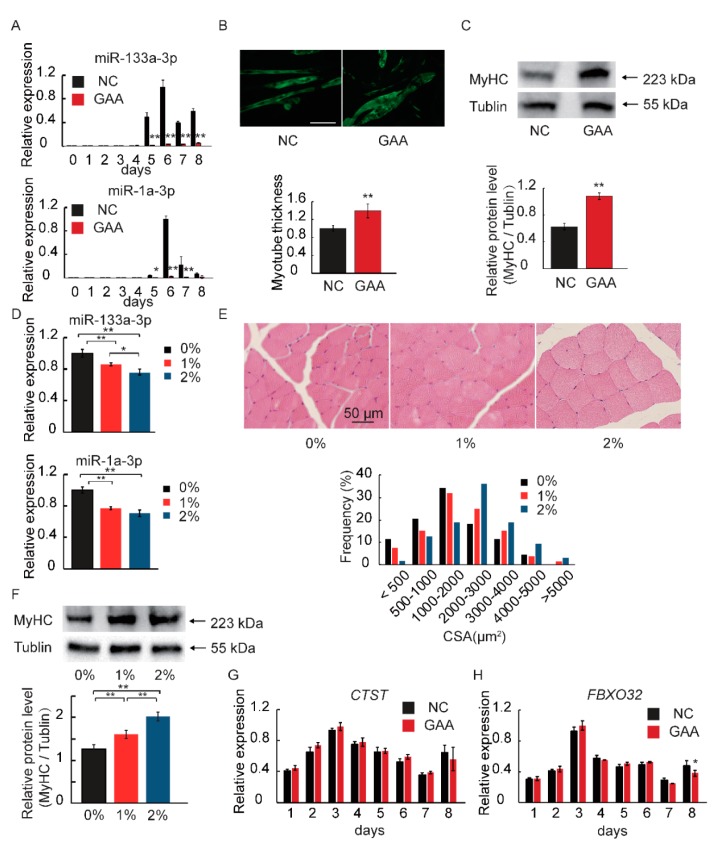
Effect of GAA on myoblasts during muscle growth. (**A**) The expression of miR-133a-3p and miR-1a-3p during myoblast differentiation after GAA treatment (Student’s *t*-test); (**B**) myotubes were stained with anti-MyHC antibody and the myotube thickness was determined (Student’s *t*-test); (**C**) MyHC protein levels were calculated by western blotting (Student’s *t*-test); (**D**) the expression of miR-133a-3p and miR-1a-3p after GAA supplement in mouse gastrocnemius tissue (Tukey’s post-hoc test); (**E**) hematoxylin-eosin staining of gastrocnemius muscle and the quantification of muscle fiber size(CSA: cross-sectional area); (**F**) MyHC protein levels were calculated by western blotting in mouse gastrocnemius tissue (Tukey’s post-hoc test); (**G**,**H**) the protein degradation marker genes (*CTSL* and *FBXO32*) were quantified by real-time PCR (Student’s *t*-test). Results are expressed as mean ± S.D. of three independent experiments. * *p* < 0.05; ** *p* < 0.01 compared with the negative control (on the bars) or between the indicated groups.

We hypothesized that miR-133a-3p and miR-1a-3p, which were the two most highly expressed in DE miRNAs, play a role in GAA-induced muscle growth. We determined the expression profiles of miR-133a-3p and miR-1a-3p throughout the myoblast differentiation phase (Figure 4A), and measured the thickness of myotubes by staining with anti-MyHC on the 6th day of myogenic differentiation (Figure 4B). The data showed a ~40% increase in the area of GAA-treated myotubes compared with that of DM myotubes (Figure 4B). In addition, the total MyHC protein level and *MyHC* isoform mRNA levels were also determined (Figure 4C and Figure S3A–C). Because cathepsin L (*CTSL*) and atrogin-1 (*FBXO32*) are marker genes of skeletal muscle atrophy [39,40], their expression was also determined, but no significant difference in this regard was identified between the GAA and control groups (Figure 4G,H). To further define the effect of miRNAs on muscle growth, the mice were treated with GAA dissolved in drinking water. We detected creatinine content in serum (Figure S1B) and determined that 1% and 2% GAA could be added in subsequent experiments. As a result, we observed that the GAA supplement reduced the expression of miR133a-3p and miR-1a-3p in mouse gastrocnemius tissue (Figure 4D). In addition, 2% GAA administration significantly increased gastrocnemius muscle fiber size (Figure 4E) (Pearson chi-square test *p* < 0.01). Meantime, the total MyHC protein level was also enhanced by the GAA supplement (Figure 4F). These results showed that GAA-treatment-induced muscle growth was accompanied by the downregulation of miR-133a-3p and miR-1a-3p.

**Figure 5:**
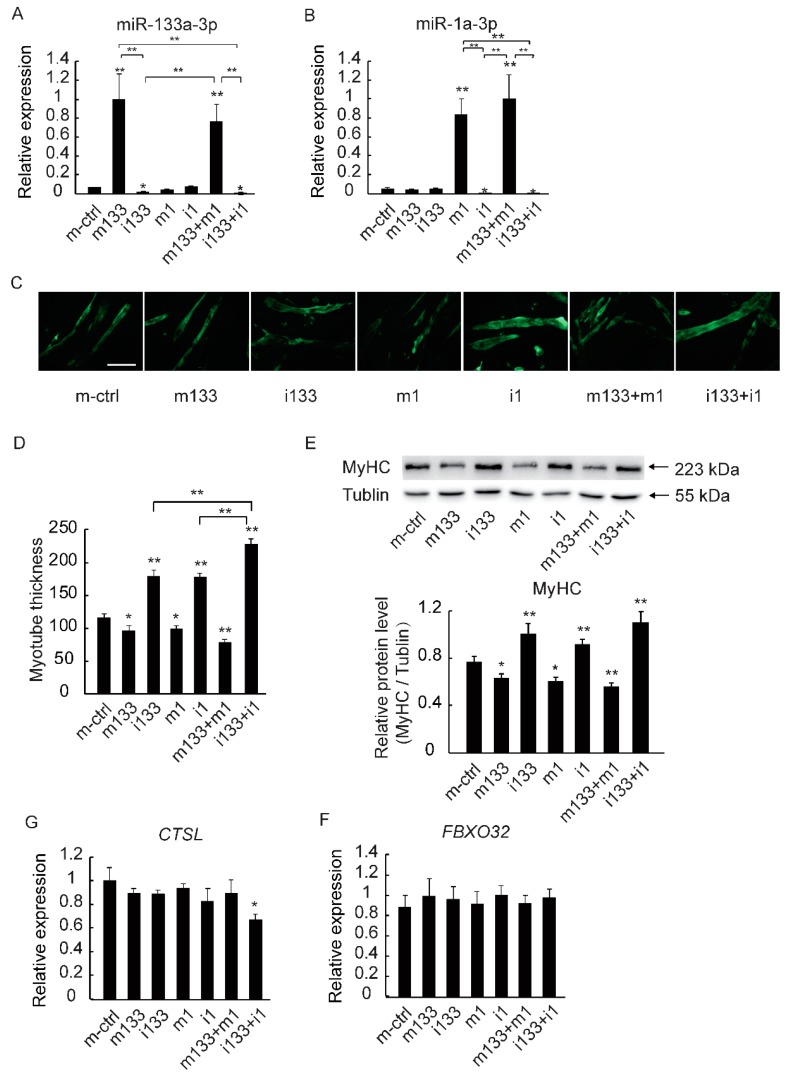
The downregulation of miR-133a-3p and miR-1a-3p promotes muscle growth. (**A**,**B**) The relative expression of miR-133a-3p and miR-1a-3p after overexpression of miRNA; (**C**,**D**) myotubes were placed in DM with and without GAA for differentiation for 6 days and then stained with anti-MyHC antibody. Myotube thickness was calculated (m-ctrl: mimic control; m133: miR-133a-3p mimic; i133: miR-133a-3p inhibitor; m1: miR-1a-3p mimic; i1: miR-1a-3p inhibitor); (**E**) the protein levels of total MyHC were calculated by western blotting after the overexpression of miRNA mimic; (**F**,**G**) the expression of marker genes of protein degradation (*CTSL* and *FBXO32*) was quantified by real-time PCR. The results are expressed as mean ± S.D. of three independent experiments. * *p* < 0.05; ** *p* < 0.01 compared with the negative control (on the bars) or between the indicated groups (Tukey’s post-hoc test).

By applying gain- and loss-of-function approaches to further verify that myotube growth was mediated by the DE miRNAs, the neo-myotubes were transfected with miR-133a-3p, miR-1a-3p, and their inhibitors, and qRT-PCR was used to detect the transfection efficiency (Figure 5A,B). In addition, *MyHC* isoform mRNA levels were also determined (Figure S3D–F). Anti-MyHC staining indicated that myotube thickness was significantly increased after the transfection of miRNA inhibitors and that the co-transfection of miR-1 and miR-133 inhibitors had a synergistic effect (Figure 5C,D).

The qRT-PCR results and western blot analysis revealed a significant increase in MyHC mRNA and protein levels in the inhibitor-transfected myotubes (particularly upon co-transfection with miR-133a-3p and miR-1a-3p inhibitors) and the MyHC levels were the highest in the group co-transfected with inhibitors (Figure 5E). Meanwhile, the expression of *CTSL* and *FBXO32* had no significant difference between the groups (Figure 5F,G). These results confirmed that the downregulation of miR-133a-3p and miR-1a-3p caused myotube growth in C2C12 cells, which resembled the effect of GAA treatment.

### 2.4. The Downregulation of MiR-133a-3p and MiR-1a-3p Promotes Muscle Growth by Activating Akt/mTOR/S6K Signaling

**Figure 6:**
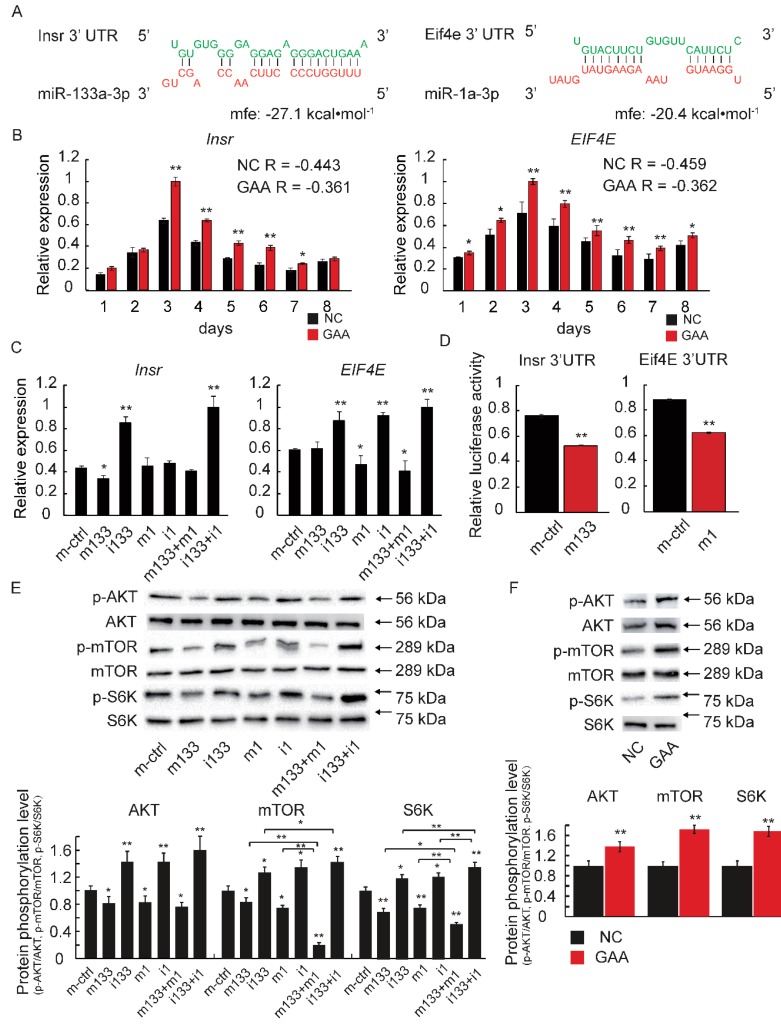
The downregulation of miR-133a-3p and miR-1a-3p promotes skeletal muscle growth by activating Akt/mTOR signaling. (**A**) Potential binding sites for miR-133a-3p and miR-1a-3p in the 3′UTR of *Insr* and *EIF4E* mRNA, respectively, predicted by RNAhybrid (MFE: minimal free energy); (**B**) the relative expression of the miRNA target gene after GAA treatment or miRNA mimic overexpression (Student’s *t*-test) (R: Pearson correlation coefficient); (**C**) the relative expression of the miRNA target gene after miRNA mimic overexpression (Tukey’s post-hoc test); (**D**) luciferase reporter assay indicated that transfection with miRNA mimic significantly suppressed the relative activity of luciferase (Student’s *t*-test); (**E**,**F**) the Akt, mTOR and S6K phosphorylation levels detected by western blotting after the overexpression of miRNA mimic or GAA treatment (Tukey’s post-hoc test). The results are expressed as mean ± S.D. of three independent experiments. * *p* < 0.05; ** *p* < 0.01 compared with negative control (on the bars) or mimic control (on the bars) or between the indicated groups.

To explore the potential signaling pathways of miR-133a-3p and miR-1a-3p, we focused on results obtained from TargetScan and RNAhybrid to explore their predicted target genes. Insulin Receptor (*Insr*) and Eukaryotic Translation Initiation Factor 4E (*EIF4E*) were predicted to be the target genes of miR-133a-3p and miR-1a-3p, respectively. Notably, the free energy score, given by RNAhybrid, was very low (−27.1 and −20.4 kcal/mol, respectively) (Figure 6A). These results suggested that miR-133a-3p and miR-1a-3p are likely to interact with the 3′UTR of *Insr* and *EIF4E* mRNA, and then post-transcriptionally downregulate its expression. To confirm this hypothesis, we performed qRT-PCR, which revealed that the expression of *Insr* and *EIF4E* was significantly elevated after GAA treatment (Figure 6B). Meanwhile, we analyzed the correlation of the expression levels of miRNAs and their target mRNAs (Figure 4A and Figure 6B). The results revealed a negative correlation between the expression of miRNAs and their target mRNAs (Figure 6B). As expected, the miRNA transfection experiment (Figure 5A,B) revealed the same tendency—the target mRNA levels were improved in the inhibitor-transfected myotubes (Figure 6C). The above data indicated that either *Insr* or *EIF4E* could be target genes for miR-133a-3p and miR-1a-3p, respectively. To further test whether miR-133a-3p and miR-1a-3p directly target the 3′UTR of *Insr* and *EIF4E* mRNA, we performed a dual-luciferase reporter assay in HeLa cells, the results of which was shown in Figure 4F. The findings revealed that miR-133a-3p significantly reduced the luciferase activity in HeLa cells transfected with the *Insr* 3′UTR reporter. Similarly, miR-1a-3p was able to reduce the luciferase activity in HeLa cells transfected with the EIF4E 3′UTR reporter (Figure 6D). These results indicated that the *Insr* and *EIF4E* genes were direct targets of miR-133a-3p and miR-1a-3p, respectively.

The target genes of miR-133a-3p and miR-1a-3p (*Insr* and *EIF4E,* respectively) play roles in the Akt/mTOR/S6K pathway, which is a crucial regulator of muscle growth and hypertrophy. Against this background, the phosphorylation levels of Akt, mTOR and S6K were determined by western blotting, with the results indicating significant increases in the Akt, mTOR and S6K phosphorylation levels with miRNA inhibitor treatment. A synergistic effect was also revealed in the co-transfection inhibitor group. However, a significant decrease in the phosphorylation of Akt, mTOR and S6K was observed in miRNA mimic-transfected myotubes (Figure 6E). In addition, the phosphorylation levels of both Akt, mTOR and S6K were also increased after GAA supplement in vitro and in vivo (Figure 6F and Figure S1C). Meanwhile, we found that rapamycin (Rap) enhanced the phosphorylation level of Akt and blocked the GAA—mediated activation of mTOR/S6K signaling (Figure S1D).

These results demonstrate that the GAA-induced downregulation of miR-133a-3p and miR-1a-3p promotes muscle growth by activating Akt/mTOR/S6K signaling.

## 3. Discussion

GAA is an endogenic amino acid derivative, and it acts as a direct precursor to Cr, which is an essential compound in energy metabolism. GAA is synthesized by the kidneys and through circulation transported to the liver for synthesis of creatine [41,42]. The finding that no GAA is detected in the human skeletal muscle after GAA supplementation is controversial [43]; however, our results have revealed that exposure of cells to 20 mM GAA-containing media can significantly increase intracellular creatine content (Figure S1A). Previous studies have shown that the specific activity of GAMT in the muscle appears to be low [44], but enough to synthesize all creatine needed in this tissue [45]. This might mean that exogenous supplement GAA can be absorbed in a small quantity and efficiently utilized by muscle tissue. Creatinine is a product of muscle metabolism in the body and expelled through circulation. Our results indicate that 2% and 3% GAA supplement could significantly increase the content of creatinine (Figure S1B). This indicates that the absorption of GAA has been overloaded.

Skeletal muscle has the ability to rapidly adapt its structure and function to external stimuli, allowing a diverse range of movements. The composition of skeletal muscle is broadly determined by regulatory systems that control the balance between muscle protein synthesis and degradation [46]. However, many external factors can disrupt this balance, including aging [47], myasthenia gravis [48] and Duchenne muscle dystrophy [49], involving the selective degradation of type II fibers. Muscle atrophy is also associated with the reduced regenerative capacity of muscle stem cells. Skeletal regeneration is a complicated process that depends on the regulation of myogenic regulatory factors (MRFs), among which MyoD initiates myogenic differentiation and myoblast formation [50], and MyoG is essential for the differentiation of most myogenic precursor cells. Our results show that GAA was able to promote myogenic differentiation by upregulated *MyoD* and *MyoG* (Figure 2D). Notably, GAA treatment increased myofiber thickness, skeletal muscle cross-sectional area and total MyHC protein levels, which can also contribute to muscle growth (Figure 4B,C,E,F). Previously, McCarthy et al. [51] reported that overload-induced hypertrophy in skeletal muscle (including soleus and plantaris) decreased the expression of miR-133a-3p and miR-1a-3p. In our study, we founded the downregulation of miR-133a-3p and miR-1a-3p in myotubes or growing skeletal muscle after GAA treatment (Figure 3C and Figure S2). Thus, GAA may contribute to skeletal muscle hypertrophy, but this needs further study due to Akt1 activation promoting the selective expression of type IIb muscle fibers [52]. Here, our results on *MyHC IIb* mRNA levels revealed that they were significantly improved by GAA treatment (Figure 3A–C). Thus, GAA may help prevent muscle loss and reduce chronic diseases and mortality in the elderly.

Next, the analyses of GO terms and KEGG pathways based on the small RNA-seq results revealed that DE miRNAs were directly or indirectly involved in myogenesis. The target genes of most of the DE miRNAs were particularly associated with “cell differentiation” and “embryo development”, which were probably related to myogenic differentiation [53,54]. The results of MyHC staining (Figure 2C) revealed that the myoblasts had a higher rate of differentiation after GAA treatment. Moreover, the target genes of some DE miRNAs that were shown to be particularly associated with “biosynthetic process” and “growth” have been studied in relation to tissue development and the biological process of protein. Our results also indicated that GAA treatment induced more MyHC-positive cells and a greater fiber diameter (Figure 4B). Notably, the KEGG pathway results showed that the target genes were particularly associated with “Wnt signaling pathway”, “PI3K–Akt signaling pathway”, and “AMPK signaling pathway”, which are thought to be typical features of muscle growth and hypertrophy [55,56,57,58]. Intriguingly, GAA may act as an activator of gamma-amino butyric acid (GABA) receptors in the brain and peripheral tissues [59]. In virtue of GABA being the principal inhibitory neurotransmitter in the nervous system and reported to be associated with food intake in pigs and rats [60,61], exogenous GAA might act as a neuromodulator and affect neuronal excitability, muscular tone, brain development, and food intake. However, the precise mechanism by which GABA receptor is activated by GAA is unclear. Here, we found that the target genes of miR-335-3p were particularly associated with “GABAergic synapse”. Thus, GAA may activate the GABA receptor via mir-335 modulation, but this needs to be proven experimentally.

Akt/mTOR/S6K signaling is a typical protein synthesis pathway. Many nutrients promote muscle cell growth or hypertrophy by activating this signaling pathway, such as alpha-ketoglutarate [62], betaine [63], black ginseng [64] and folic acid [65]. It has been reported that insulin can also promote muscle growth [66]; our study also demonstrates that GAA promotes skeletal muscle growth through *Insr*/AKT/mTOR signaling (Figure 6F and Figure S1C). However, notably, previous studies suggested that exogenous GAA stimulates insulin secretion in rats [67]. In other words, GAA could also activate insulin-related signaling pathways in the absence of insulin by miRNA. What is noteworthy is that EIF4E is downstream of the Akt/mTOR signaling pathway, but the miR-1a-3p inhibitor was also shown to be capable of increasing the phosphorylation levels of Akt, mTOR and S6K. This was because miR-1a-3p has other target genes that contributed to the Akt/mTOR/S6K pathway, such as Epha2, Lpar5, HSP90b1 and Ywhab (Table S2). Rap is an inhibitor of mTOR signaling [68]. Our results demonstrated that GAA-induced activated mTOR/S6K signaling can be blocked by Rap supplement (Figure S1D). Meantime, the enhanced effect of Rap in activating Akt phosphorylation level was also observed (Figure S1D), consistent with previous research [69].

## 4. Materials and Methods

### 4.1. Regents and Antibodies

GAA (G11608) and Rapamycin (Rap) (V900930) were purchased from Sigma-Aldrich (St. Louis, MO, USA). Dimethyl sulfoxide (DMSO) was purchased from Solarbio Science Technology Co. (Beijing, China); Antibodies fast-MyHC (ab51263), phosphor-Akt (ser473) (ab81283), Akt (ab179463), phosphor-mTOR (ser2448) (ab10926), mTOR (ab134903), phosphor-S6K (Thr389) (ab2571) and S6K (ab186753) were purchased from Abcam (Cambridge, MA, USA). Antibody against tublin (#2148) were purchased from Cell Signaling Biotechnology (Beverly, MA, USA).

### 4.2. Cell Culture and Treatment

The murine skeletal muscle cell line C2C12 was cultured in growth medium (GM) that contained high-glucose DMEM (Hyclone, Logan, UT, USA), supplemented with 10% fetal bovine serum (GIBCO, Grand Island, NY, USA), 100,000 units/L penicillin sodium, and 100 mg/L streptomycin sulfate (Hyclone) at 37 °C in a humidified atmosphere containing 5% CO_2_. The C2C12 myoblasts were induced with differentiation medium (DM) containing high-glucose DMEM and 2% horse serum (GIBCO, Grand Island, NY, USA) for 8 days. GAA was soluble in GM or DM at a final concentration of 20 mmol/L. Rapamycin (Rap) was diluted in DMSO at a final concentration of 100 nmol/L; the final concentration of DMSO was less than 0.1% in the culture.

### 4.3. Animals and Muscle Collection

The Kunming male mice (*n* = 30) at 3 weeks of age were purchased from DASHUO Medical Laboratory Animal Center (Chengdu, China). The mice were given free access to food and water under constant 12 h light and 12 h dark cycle at a temperature of 23 °C ± 3 °C and relative humidity of 70% ± 10% throughout experimental period. After one week of acclimatization, the mice were randomly divided into three groups (*n* = 10 in each group) and supplemented with different concentrations of GAA (0%, 1% and 2%), dissolved in drinking water for 8 weeks. At the end of the experiment, serum was collected and the gastrocnemius muscle was taken for further testing. The serum was stored at −80 °C, the muscle stored in liquid nitrogen.

### 4.4. RNA Extraction, Reverse Transcription, and qRT-PCR 

Total RNAs were extracted from C2C12 myotubes using RNAiso Plus reagent (Takara, Guangdong, China), in accordance with the manufacturer’s instructions. For mRNA, total RNA (2 µg) was reverse-transcribed to cDNA in a final volume of 20 µL using PrimeScript™ RT Reagent Kit (Takara, Guangdong, China). For MicroRNA, reverse transcripts were synthesized using Mir-X™ miRNA First-Strand Synthesis Kit (Clontech, Mountain View, CA, USA). GAPDH or U6 was used as a candidate housekeeping gene for mRNA and miRNA, respectively. TB Green™ Premix Ex Taq reagents (Takara, Guangdong, China) and sense and antisense primers (200 nM for each gene) were used for quantitative real-time polymerase chain reaction (qRT-PCR). PCR reactions were performed using the CFX Connect Real-Time System (BIO-RAD, Hercules, CA, USA). Primer sequences are presented in Table S1.

### 4.5. Cell Proliferation Ratio Assay

C2C12 cells proliferation ratio was detected by Cell Counting Kit-8 (CCK-8) (Beyotime, Shanghai, China) and EdU Cell Proliferation Assay Kit (Ribobio, Guangzhou, China). C2C12 cells were seeded in a 96-well cell culture plate and maintained in GM, which contained 0 mM, 5 mM, 10 mM or 20 mM GAA for 48 h; the medium was changed every 12 h. For CCK-8, 10 μL CCk-8 reagent was added to the medium, after 1 h, optical density was measured at 450 nm. For EdU cell proliferation assay, cells were treated with GAA of different concentrations, and when the cells reached approximately 80% confluence, 10 μM EdU was added to the medium and incubated for 2 h. EdU staining was done according to the manufacturer’s instructions; cell nucleus was stained by DAPI (Beyotime, Shanghai, China). Stained cells were imaged by Olympus IX53 microscope (Olympus, Totyo, Japan).

### 4.6. Western Blot Assay

Western blot was performed as previously described [70]. Briefly, the cells were seeded in a six-well cell culture plate. After differentiation for 4 days, cells were incubated with Rap or GAA as described for 48 h. The cells were then rinsed in PBS three times and lysed in 200 μL RIPA lysis buffer that contained 1 mM PMSF and 0.02% Protease phosphatase inhibitors. For tissues, 25 mg gastrocnemius muscle was homogenized in 500 μL RIPA lysis buffer that contained 1 mM PMSF and 0.02% Protease phosphatase inhibitors. Afterwards, the homogenized liquids were shocked for 30 min at 4 °C, and the insoluble matter was removed from the suspension by 12,000× *g* centrifugation for 15 min. The total protein concentration was quantified using BCA protein assays. After aliquots 50 μg protein suspension was separated by 10% sodium dodecyl sulfate (SDS) polyacrylamide electrophoresis gels, the proteins were transferred to polyvinylidene fluoride (PVDF) membranes (BIO-RAD, Hercules, CA, USA) using the Trans-Blot Turbo transfer system (BIO-RAD, Hercules, CA, USA), and then blocked with 5% (*w*/*v*) non-fat dry milk in Tris-buffered saline that contained 0.1% Tween 20 (BIO-RAD, Hercules, CA, USA) for 2 h at room temperature. The PVDF membranes were then incubated with the indicated antibodies at 4 °C overnight, followed by incubation with the secondary antibody for 1 h at room temperature. Protein expression was measured using a ChemiDoc MP Imaging System (BIO-RAD, Hercules, CA, USA) and normalized to tubulin expression.

### 4.7. Immunocytochemistry

The C2C12 cells were rinsed three times in PBS and washed in 0.3% Triton X-100 for 30 min, followed by blocking for 1 h at room temperature. Subsequently, these cells were incubated overnight in mouse anti-fast-MyHC at 4 °C. The next day, the C2C12 cells were incubated with Alexa Fluor^®^ 488 secondary antibody (ab150113; Abcam) for 1 h, and the cell nucleus was stained by DAPI (Beyotime, Shanghai, China). The cells were observed, and the fluorescence was quantified using Olympus IX53 microscope (Olympus, Totyo, Japan) with cellSens Standard software (v1.16, Olympus Instruments, Totyo, Japan). Up to six fields of view were captured from each group.

### 4.8. HE Staining and Skeletal Muscle Cross-Sectional Area Statistics

Hematoxylin and eosin (HE) staining was carried out for the gastrocnemius muscle. In detail, the gastrocnemius muscle from 0%, 1% and 2% GAA supplemented mice were fixed in 4% paraformaldehyde at room temperature for 30 min, then dehydrated in paraffin. Sections were prepared and stained by HE Staining Kit (Beyotime, Shanghai, China) according to the manufacturer’s instructions; the images were captured by Olympus IX53 microscope (Olympus, Totyo, Japan). Skeletal muscle size was quantified by cellSens Standard software (Olympus Instruments); 300 fibers were measured per muscle tissue.

### 4.9. Small RNA Library Construction and Sequencing

Total RNA of C2C12 on the sixth day of differentiation was extracted by RNAiso Plus reagent. RNA concentration and quality were examined using Agilent 2100 Bioanalyzer and Agilent RNA 6000 Nano Kit (Agilent Technologies, Santa Clara, CA, USA); the sample with RIN (RNA Intergrity Number) under 8 were excluded from the following processes. Small RNAs ranging from 14 to 36 nt in size were purified by polyacrylamide gel electrophoresis. To construct a small RNA sequencing library, 3′ and 5′ adaptors were ligated with small RNA fractions. The modified small RNA was then reverse-transcribed and amplified by reverse-transcription PCR. Finally, the enriched cDNA was sequenced on an Illumina HiSeq 2500, in accordance with the manufacturer’s instructions. The raw sequencing data have been deposited in NCBI’s Gene Expression Omnibus and are accessible through GEO Series accession number GSE118528.

### 4.10. Analysis of High-Throughput Sequencing Data

The raw reads were subjected to a series of additional filters, such as removing low-quality reads, repeated sequences, adaptor sequences, and reads shorter than 14 nt or longer than 36 nt. The remaining sequences were defined as clean reads and processed using miRDeep2 software. Briefly, the clean reads were mapped to a reference mouse genome (assembly GRCm38.p5). All mappable reads were used as queries for searches against the known mouse miRNA precursors and mature miRNAs in the miRbase database 21.0, to identify known mouse miRNAs. The length distribution of the clean reads was generated. Expression of identified known mouse miRNAs were achieved by normalizing read counts to RPM formula: Normalized expression = (actual miRNA sequencing read count/total mappable read count) × 1,000,000 for further analysis. More details about raw data are presented in Table S2.

### 4.11. Differential Expression Analysis of MiRNAs

To identify Differential Expression (DE) miRNAs, read counts of DE miRNAs were used for differential expression analysis using edgeR v3.7 [71] in OmicShare tools (www.omicshare.com/tools) between the control group and the GAA group. A unique miRNA was considered to be differentially expressed when it met the following criteria: |log2 (fold change)| > 1 and false discovery rate (FDR) < 0.01.

### 4.12. Prediction and Functional Enrichment Analysis of DE MiRNA Target Genes

To further understand the function of DE miRNA, we predicted DE miRNA target genes using TargetScan 7 [72] (Table S3). The Gene Ontology (GO) terms and Kyoto Encyclopedia of Genes and Genomes (KEGG) pathway terms that were strongly associated with the predicted target genes were identified using Database for Annotation, Visualization and Integrated Discovery (DAVID) (v6.8, Frederick, MD, USA) [73]. The target genes list was uploaded into the DAVID software, and the species and the background was selected as “Mus musculus”. The results were shown by −log_10_ *p* value.

### 4.13. Bioinformatic Analyses and Dual-Luciferase Reporter Assay 

miRNA sequence information was obtained from miRBase (http://www.mirbase.org/search.shtml). Target predictions were performed with miRDB (http://mirdb.org/miRDB/) [74]. MiRNA binding sites within mRNA 3′UTRs were analyzed with TargetScan (http://www.targetscan.org/vert_71/). The minimum free energy of the hybridization of mRNA 3′UTR and miRNA was predicted using RNAhybrid (https://bibiserv.cebitec.uni-bielefeld.de/rnahybrid) [75]. When the density of Hela in a 48-well plate reached 70%, the potentially targeted mRNAs containing miRNA binding sites were cloned into the pmirGLO plasmid (Promega, Madison, WI, USA), and co-transfected with miRNA mimic into Hela cells using Lipofectamine 3000 (Invitrogen, Grand Island, NY, USA) according to the manufacturer’s instructions. Further culture for 48 h and dual-luciferase activity was quantitated by using the Dual-Luciferase Reporter Assay System kit (Promega, Madison, WI, USA) according to the manufacturer’s instructions.

### 4.14. MiRNA Transfection

The specific miRNA mimic, inhibitor, and mimic control were purchased from RIBOBIO (Guangzhou, China). When C2C12 was differentiated four days later, the cells were transfected with mimic control (miRNA mimic that contains no sequence similarity to any reported mice gene sequence), miRNA mimic, and inhibitor (transfected with a single RNA sequence exactly complementary to the specific miRNA mimic) by using Lipofectamine 3000 (Invitrogen, Grand Island, NY, USA) according to the manufacturer’s instructions.

### 4.15. Creatine and Creatinine Content Assay

The content of creatine and creatinine were evaluated by using the Creatine assay kit (BioAssay Systems, The Bay Area, CA, USA) and creatinine assay kit (BioAssay Systems, The Bay Area, CA, USA), respectively. Briefly, for creatine detection, C2C12 was cultured in GM, which contented 20 mM GAA, for 24 h. The cells were then collected in a 1.5 mL centrifuge tube using Trypsin-EDTA Solution (GIBCO, Grand Island, NY, USA) and 1000 rpm centrifugation for 5 min. 20 μL ultrapure water was then added, the cell membranes were broken through ultrasound, and the insoluble matter was removed from the suspension by 12,000× *g* centrifugation for 15 min. The creatine concentration was quantified using a Creatine assay kit in accordance with the manufacturer’s instructions. For creatinine detection, the mice were fed for 1 week and serum was extracted to determine creatinine content by using the Creatinine assay kit, in accordance with the manufacturer’s instructions.

### 4.16. Statistical Analysis

The data are expressed as the mean ± standard deviation (SD) of three independent experiments. Statistical significance was calculated using the Student’s *t*-test for comparisons of two groups, and one-way analysis of variance with Tukey’s post-hoc test for multiple groups using SPSS 19.0 software (SPSS Inc., Chicago, IL, USA). A value of *p* < 0.05 was considered statistically significant (* *p* < 0.05, ** *p* < 0.01).

## 5. Conclusions

Our investigations suggest that GAA could effectively promote C2C12 myoblast differentiation and muscle growth and modulate myomiRs, including miR-133a-3p and miR-1a-3p. Further experimentation demonstrated that GAA promotes myoblast differentiation and muscle growth by downregulating miR-133a-3p and miR-1a-3p and activating Akt/mTOR/S6K signaling. Our findings suggest that GAA enhances muscle performance, which may represent a promising therapeutic route for muscle atrophy and related disorders.

## Supplementary Materials

Supplementary materials can be found at http://www.mdpi.com/1422-0067/19/9/2837/s1.
