# Supplementary material for: Guanidinoacetic Acid Regulates Myogenic Differentiation and Muscle Growth Through miR-133a-3p and miR-1a-3p Co-mediated Akt/mTOR/S6K Signaling Pathway
Source: Int J Mol Sci. 2018 Sep 19;19(9):2837. doi: 10.3390/ijms19092837 (PMC6163908; doi:10.3390/ijms19092837)
Supplement: Supplementary file 1 [file ijms-19-02837-s001.zip › ijms-352774-supplementary.pdf]

Supplementary Materials

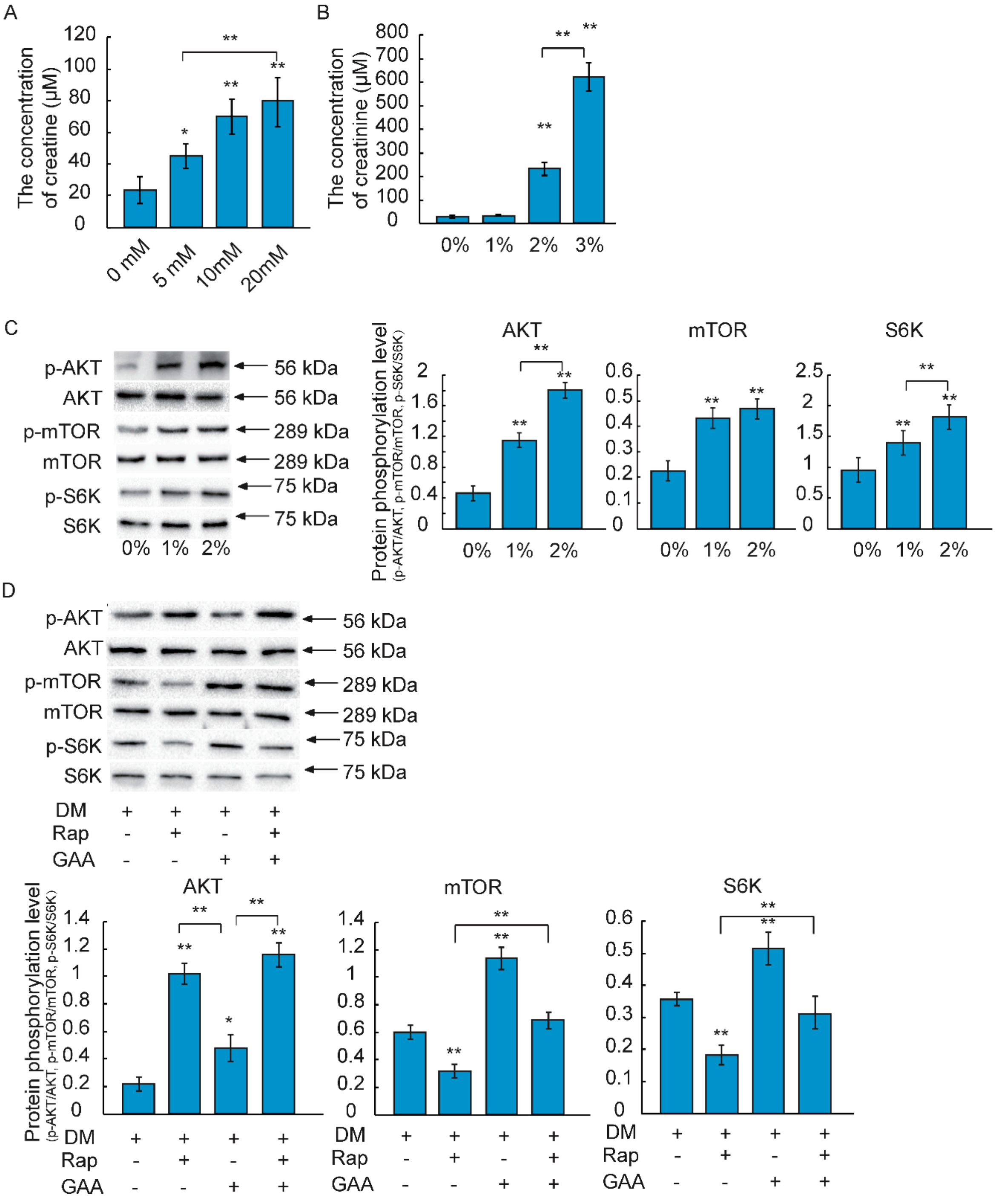

Figure S1. GAA activated AKT/mTOR/S6K signaling pathway. (**A**) The concentration of creatine in the C2C12 treated 20 mM GAA for 24 h; (**B**) the concentration of creatinine in the serum of mice, which treated with different concentration of GAA for 1 W; (**C**) the Akt, mTOR and S6K phosphorylation levels detected by western blotting *in vivo*; (**D**) the Akt, mTOR and S6K phosphorylation levels detected by western blotting after myotubes treated with rapamycin (Rap) and GAA. The results are expressed as mean ± S.D. of three independent experiments. *$p < 0.05$, **$p < 0.01$ compared with the negative control (on the bars) or between the indicated groups (Tukey's post-hoc test).

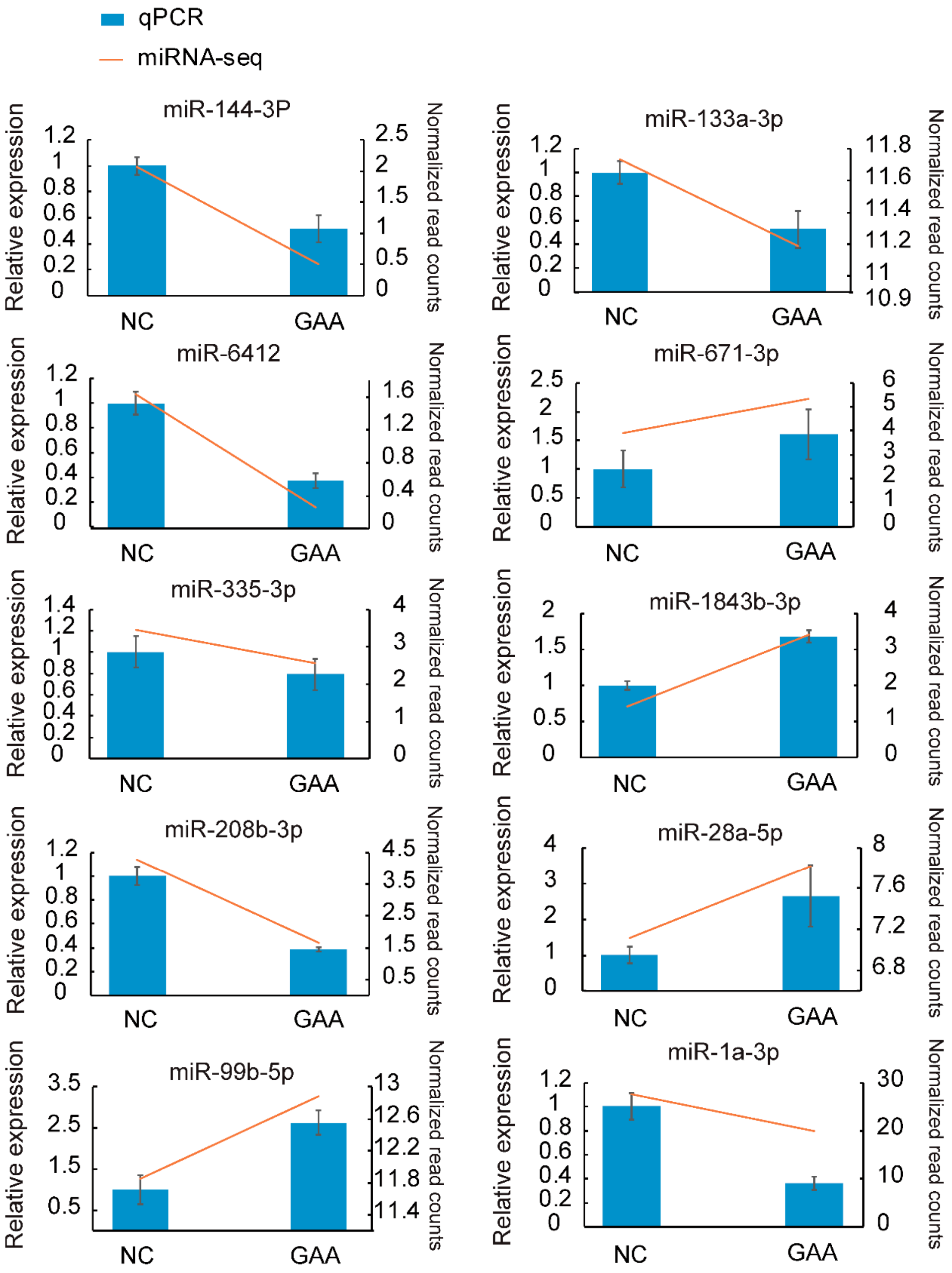

Figure S2. qRT-PCR validation of 10 miRNAs. Lines symbols represent the miRNA sequencing reads (normalized), bars represent the miRNA relative expression abundance determined by qRT-PCR. The results are expressed as mean ± S.D.

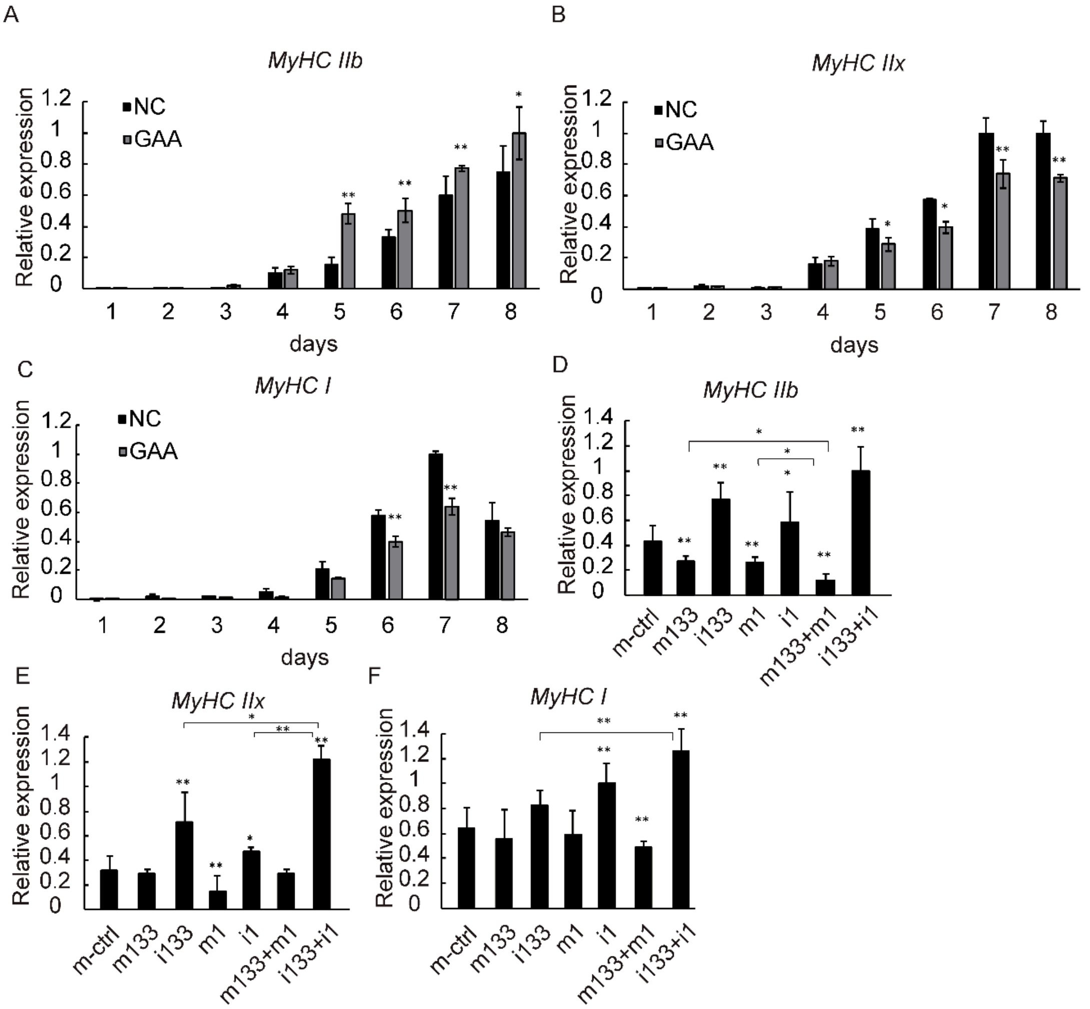

Figure S3. The relative expression of MyHC isoform. The results are expressed as mean ± S.D. of three independent experiments. **p* < 0.05, ***p* < 0.01 compared with the negative control or mimic control (on the bars) or between the indicated group (Student's *t*-test for A, B and C; Tukey's post-hoc test for D, E and F).
