# Supplementary figures and images for: Guanidinoacetic Acid Regulates Myogenic Differentiation and Muscle Growth Through miR-133a-3p and miR-1a-3p Co-mediated Akt/mTOR/S6K Signaling Pathway
Source: Int J Mol Sci. 2018 Sep 19;19(9):2837. doi: 10.3390/ijms19092837 (PMC6163908; doi:10.3390/ijms19092837)

A

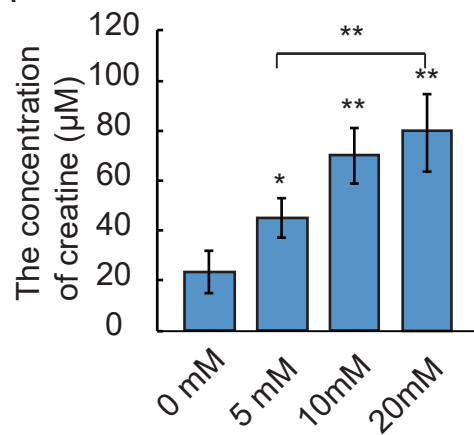

B

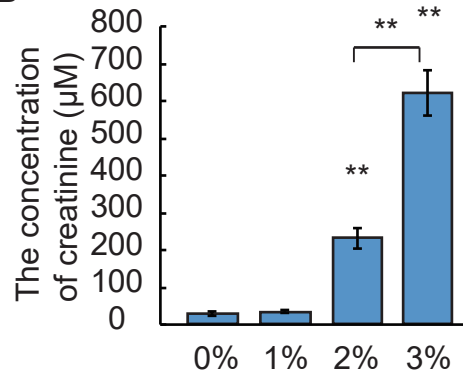

C

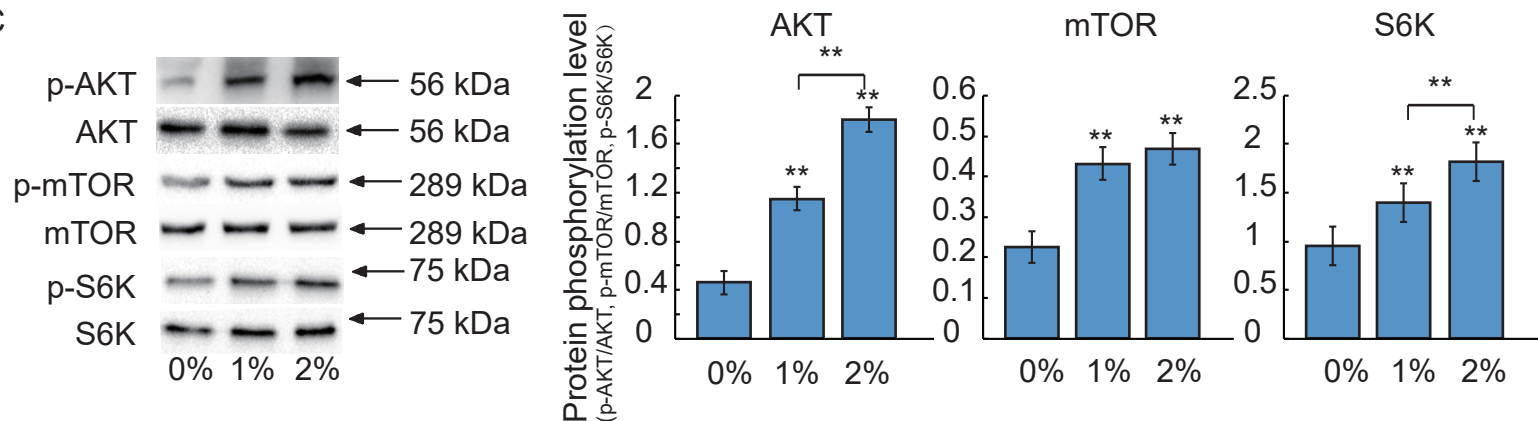

D

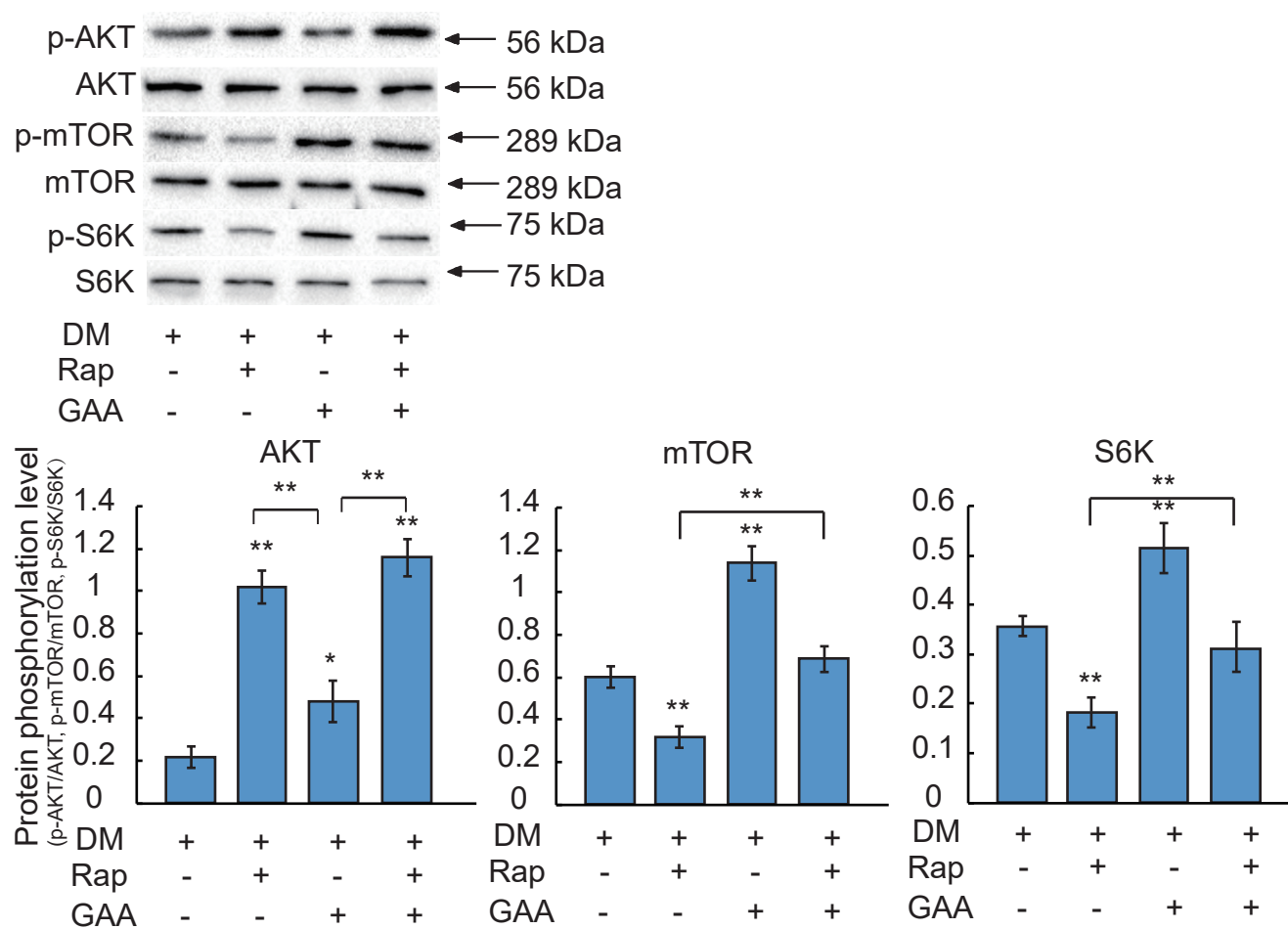

Supplement: Supplementary file 1 [file ijms-19-02837-s001.zip › Figure S1.pdf]

■ qPCR  
— miRNA-seq

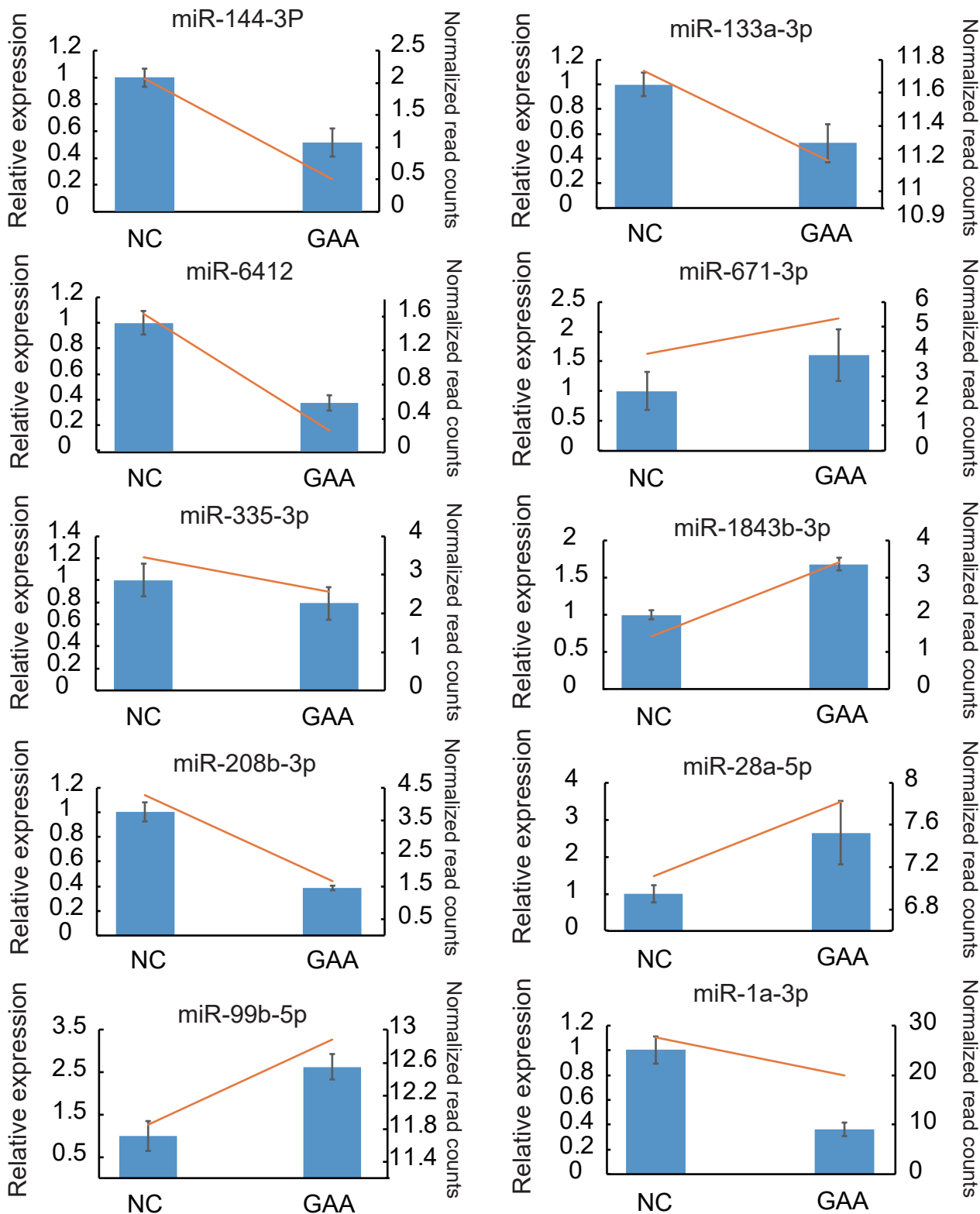

Supplement: Supplementary file 1 [file ijms-19-02837-s001.zip › Figure S2.pdf]

A

*MyHC IIb*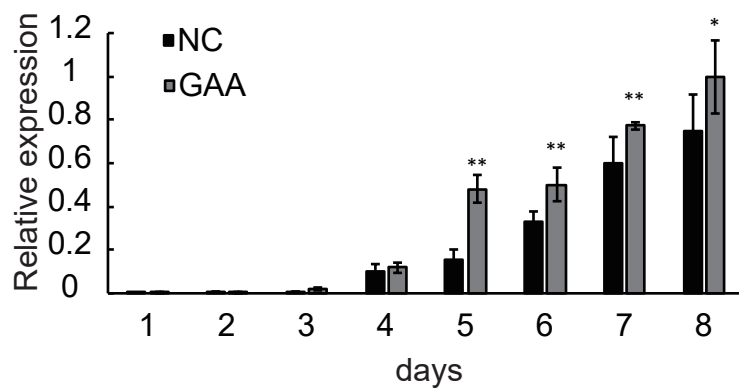

B

*MyHC IIx*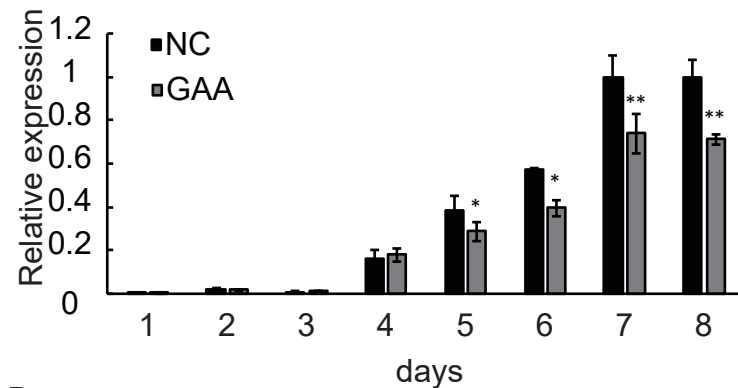

C

*MyHC I*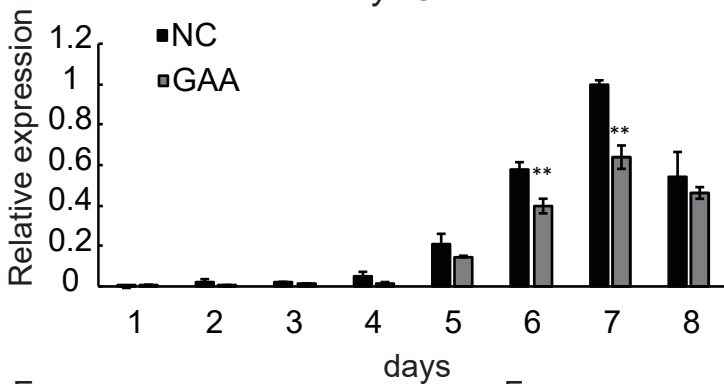

D

*MyHC IIb*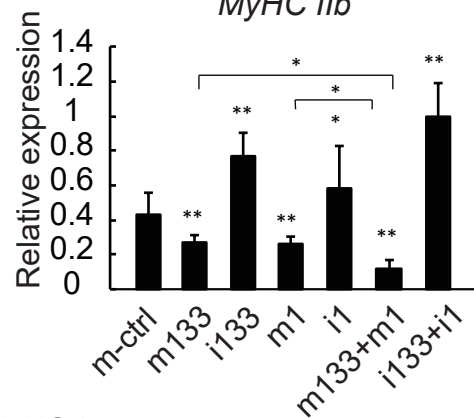

E

*MyHC IIx*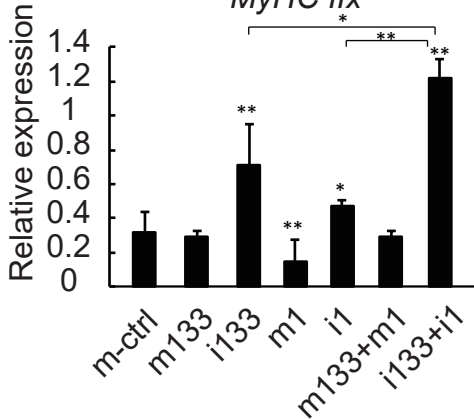

F

*MyHC I*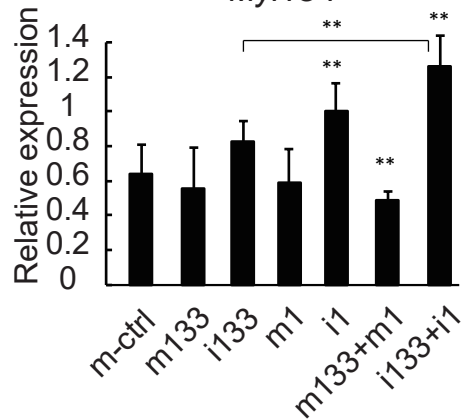

Supplement: Supplementary file 1 [file ijms-19-02837-s001.zip › Figure S3.pdf]
